# Somatosensory Cross-Modal Reorganization in Adults With Age-Related, Early-Stage Hearing Loss

**DOI:** 10.3389/fnhum.2018.00172

**Published:** 2018-05-03

**Authors:** Garrett Cardon, Anu Sharma

**Affiliations:** ^1^Department of Psychiatry, University of Colorado Denver Anschutz Medical Campus, Aurora, CO, United States; ^2^Department of Speech, Language, and Hearing Sciences, University of Colorado Boulder, Boulder, CO, United States

**Keywords:** neuroplasticity, cross-modal plasticity, sensorineural hearing loss, sLORETA, mild-moderate hearing loss, age-related hearing loss, somatosensory evoked potentials

## Abstract

Under conditions of profound sensory deprivation, the brain has the propensity to reorganize. For example, intact sensory modalities often recruit deficient modalities’ cortices for neural processing. This process is known as cross-modal reorganization and has been shown in congenitally and profoundly deaf patients. However, much less is known about cross-modal cortical reorganization in persons with less severe cases of age-related hearing loss (ARHL), even though such cases are far more common. Thus, we investigated cross-modal reorganization between the auditory and somatosensory modalities in older adults with normal hearing (NH) and mild-moderate ARHL in response to vibrotactile stimulation using high density electroencephalography (EEG). Results showed activation of the somatosensory cortices in adults with NH as well as those with hearing loss (HL). However, adults with mild-moderate ARHL also showed robust activation of auditory cortical regions in response to somatosensory stimulation. Neurophysiologic data exhibited significant correlations with speech perception in noise outcomes suggesting that the degree of cross-modal reorganization may be associated with functional performance. Our study presents the first evidence of somatosensory cross-modal reorganization of the auditory cortex in adults with early-stage, mild-moderate ARHL. Our findings suggest that even mild levels of ARHL associated with communication difficulty result in fundamental cortical changes.

## Introduction

Age-related hearing loss (ARHL) is estimated to be the third most commonly reported chronic condition in the United States (Masterson et al., 2016). With the increase in the number of older adults and overall life expectancy (Christensen et al., 2009; Kochkin, 2009), more individuals will be at risk for acquiring ARHL than ever before. For instance, *the United States National Institute of Health-National Institute of Deafness and Other Communication Disorders* estimates that approximately 25%–45.6% of adults age 65–74 years have a disabling hearing loss (HL). This estimate increases to 78%–80% in those who are 75 years and older (Lin et al., 2011c). However, currently there is a dearth of available information regarding neuroplastic changes in ARHL.

Long-term profound sensory deprivation (i.e., as in deafness or blindness) has the potential and the tendency to lead to neuroplastic reorganization of the cerebral cortex—both between and within sensory modalities (Bavelier and Neville, 2002; Doucet et al., 2006; Mitchell and Maslin, 2007; Strelnikov et al., 2013; Glick and Sharma, 2017). For example, intact sensory systems can recruit and repurpose deprived sensory cortices for processing of their own input—a process known as cross-modal reorganization. Evidence of cortical cross-modal reorganization has been demonstrated in profound sensory insult, such as deafness and blindness, in both humans and animals (Hyvärinen et al., 1981; Sadato et al., 1996; Levänen et al., 1998; Armstrong et al., 2002; Baldwin, 2002; Auer et al., 2007; Sharma et al., 2007; Allman et al., 2009; Meredith and Lomber, 2011; Karns et al., 2012; Glick and Sharma, 2017). For instance, deaf adults have exhibited cross-modal reorganization of the auditory cortices by both the visual and somatosensory cortices (for a review see Merabet and Pascual-Leone, 2010). Similar results have been shown in deaf adults following cochlear implantation (Sandmann et al., 2012; Chen et al., 2016).

Despite the aforementioned research performed with individuals with profound sensory insult, very little is known about the effects of lesser degrees of sensory deficiency on cortical organization and plasticity. Recent studies have presented evidence of cross-modal reorganization between the visual and auditory systems in adults with mild-moderate HL (Campbell and Sharma, 2014; Stropahl and Debener, 2017). In addition, several animal studies have reported evidence of somatosensory cross-modal reorganization in deaf animals (Allman et al., 2009; Meredith and Lomber, 2011; Meredith and Allman, 2012, 2015; Meredith et al., 2012; Basura et al., 2015). For instance, Allman et al. (2009) presented evidence of somatosensory cross-modal reorganization of the auditory system in ferrets with adult onset profound deafness. Meredith et al. (2012) also presented evidence of somatosensory to auditory cross-modal reorganization in ferrets with adult-onset, partial HL (see also Schormans et al., 2017). On the other hand, no attempts have been made to investigate cortical reorganization between the auditory and somatosensory systems in humans with mild forms of HL. Finally, evidence from animal studies, which describe established anatomical connections between auditory and somatosensory cortices, suggest that the somatosensory system is a potential candidate for cross-modal interaction with the auditory system (Schroeder et al., 2001; Hackett et al., 2007).

We used high-density electroencephalography (EEG) to record cortical somatosensory evoked potentials (CSEP) in response to vibrotactile stimuli in adults with mild-moderate HL and in age-matched normal hearing (NH) controls. The aim of our study was to determine whether adults with age-related, mild-moderate HL showed evidence of somatosensory cross-modal reorganization and whether such reorganization was related to functional performance on a clinical test of speech perception in noise.

## Materials and Methods

### Subjects

The participants for the current study consisted of 19 adults between the ages of 49 and 77 years. These subjects were separated into two groups (i.e., NH and HL based on results of a comprehensive audiological evaluation: (1) NH (*n* = 9; mean age = 59.89, S.D. = 6.9); and (2) HL (*n* = 10; mean age = 66.6, S.D. = 7.3). The difference in age between these two groups was not significant (*p* > 0.05; *F* = 4.17).

**Figure 1:**
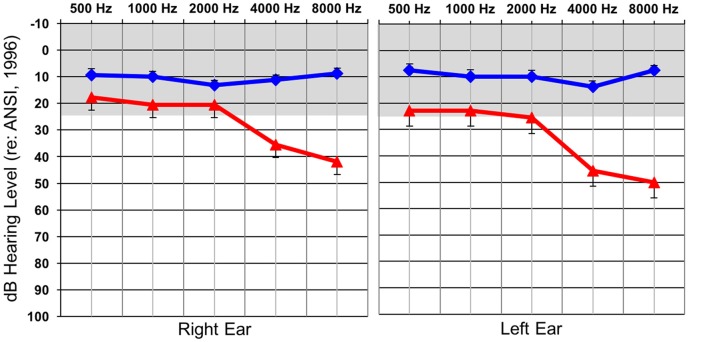
Average hearing thresholds (dBHL; y-axis) at various frequencies (x-axis) for both the normal hearing (NH; blue) and hearing loss (HL; red) groups for the right and left ear. The gray band represents the range of NH. Standard errors are shown.

Behavioral auditory thresholds were obtained by performing standard clinical behavioral audiometry with each individual. NH was defined as behavioral auditory thresholds less than or equal to 25 dBHL at 500, 1000, 2000, 4000, and 8000 Hz, while thresholds at any of these frequencies greater than 25 dBHL classified participants as having a HL. As a group, the participants in the HL group had normal behavioral auditory thresholds from 500–2000 Hz and then exhibited a sloping mild-moderate sensorineural HL between 2000–8000 Hz. Average audiometric thresholds are presented for each group in Figure 1 and reflect the classic profile of early-stage ARHL or “presbycusis”. Many of the individuals in the HL group were not aware that they had a HL at the time of testing, though some suspected that this was the case. In all instances of newly identified HL subjects were counseled by a certified clinical audiologist and were provided contact information for local audiology clinics in the event that they wanted to seek intervention. None of the participants reported having a history of neurologic diagnoses or prior intervention of their hearing losses.

### Speech Perception in Noise

The QuickSIN test, a clinical measure of auditory threshold for sentences in background noise, was used to determine acuity of speech perception in background noise (Killion et al., 2004). Stimuli were presented via a speaker placed at 0° azimuth. Standard clinical testing procedures were used: listeners were instructed to repeat two sentence lists, consisting of six sentences each, presented at 65 dB HL. Background noise was increased for each consecutive sentence in 5 dB increments, so that the signal-to-noise ratio (SNR) began at 25 dB and ended at 0 dB for the last sentence. The SNR score from the two lists was averaged for each listener, providing the level necessary for each individual to correctly repeat 50% of the key words in each sentence. The lower the SNR score, the greater the level of background noise that could be tolerated by the listener, and the better the performance.

### EEG Recording and Initial Processing Procedures

High-density EEG was performed with each participant. Specifically, subjects were fitted with a 128-electode net (Electrical Geodesics Inc., Eugene, OR, USA) and were seated comfortably in an electrically shielded sound-treated both. Continuous EEG was collected via Netstation 4 software (Electrical Geodesics Inc.) in response to a vibrotactile stimulus that was applied to the subjects’ right index finger using E-Prime 2.0 (Psychology Software Tools, Pittsburg, PA, USA). Continuous EEG files were divided into CSEP epochs post-recording (see “EEG Analysis” section below). The sampling rate of the EEG recordings was 1 kHz and a band-pass filter set to 0.1–200 Hz was used for initial data filtering.

The stimulus was a 250 Hz tone, which was 90 ms in duration with 10 ms linear ramps at onset and offset. The stimuli were presented through a vibrotactile oscillator (Sensory Systems d.b.a. Radioear Inc., New Eagle, PA, USA; B71 Bone Transducer) that was temporarily attached to participants’ right index finger using medical tape. All stimuli were presented at a level of 55 dBHL, which results in approximately 0.122 g (1.2 m/s^2^) of acceleration vibrotactile output by the oscillator. This level was sufficient for somatosensation, but did not cause discomfort (Weinstein, 1968). Continuous white noise was presented simultaneously with the somatosensory stimulation through a loud speaker oriented at approximately 45° azimuths on the right side of participants. This noise served to mask any auditory signal produced by the oscillator in addition to the vibrotactile stimulation (Yamaguchi and Knight, 1991). Since this noise was ongoing, not time locked to the somatosensory stimulus, and was random in nature, any auditory response to it was eliminated from the time-locked CSEP recordings during evoked potential averaging (Eggermont, 2007). All participants indicated that while they could clearly feel the vibrotactile stimuli on their fingertip, they could not hear any tonal auditory signal coming from the oscillator (i.e., due to the masking sound of the white noise), consistent with previous studies (e.g., Bolognini et al., 2010). EEG data corresponding to approximately 1000 stimuli were collected for each subject (Hämäläinen et al., 1990).

Following recording, continuous EEG was segmented with respect to each stimulus. Segments consisted of 100 ms pre-stimulus and 595 ms post-stimulus. Baseline correction relative to the pre-stimulus interval was performed. Additionally, EEG epochs that contained data that were found to be ±100 μV at selected eye channels (i.e., artifactual eye-blinks) were removed. Bad channels were flagged, removed and replaced with interpolated data from remaining electrodes using a spline interpolation algorithm. The following components were observed in CSEP waveforms: P50, N70, P100, N140a, N140b—consistent with previous studies (Johnson et al., 1980; Hämäläinen et al., 1990; Bolton and Staines, 2014).

### Cortical Source Localization Analysis (Current Density Reconstruction)

Following initial CSEP waveform processing, EEG data were imported into the EEGLAB toolbox (Delorme and Makeig, 2004) working in concert with Matlab (MatLab (Version R2014b) [Software] (2014), Natick, MA, USA) in order to prepare them for cortical source localization analysis. Within EEGLAB, data were first baseline corrected relative to the pre-stimulus interval. Then, all EEG epochs that contained artifact exceeding ±100 μV were rejected. Additionally, the sampling rate of the EEG signals was changed to 250 Hz to improve speed and ease of subsequent processing. Once data had undergone these steps, they were subjected to independent components analysis (ICA) within EEGLAB. This analysis provided the consequent ability to identify and reject (i.e., prune) independent components that contained artifact and did not account for a significant portion of the variability in the EEG signal. This pruning process was repeated for each CSEP component within each subject’s EEG recording. Previous studies have shown this method to be an effective means to rid EEG recordings of spurious data prior to cortical source localization methods (Makeig et al., 1997, 2004; Hine and Debener, 2007; Debener et al., 2008; Gilley et al., 2008; Campbell and Sharma, 2014; Sharma et al., 2015).

Once pruning had occurred, data were transferred to the CURRY Scan 7 Neuroimaging Suite (Compumedics Neuroscan™, Charlotte, NC, USA) where current density reconstruction (CDR) took place. First, grand averaging of pruned EEG data for each group (i.e., NH and HL) was performed. Following this step, a second ICA was performed in order to determine the components that contained data with the highest SNR, which were to be included in the cortical source estimations. Then, a head model was created and standardized using the boundary element method (BEM; Fuchs et al., 2002; Hallez et al., 2007). CDRs were then performed via Standardized Low Resolution Brain Electromagnetic Tomography (sLORETA; Pascual-Marqui, 2002). This analysis represents the estimation of the sources of electric neuronal activity distribution (current density vector field; Pascual-Marqui, 2002, 2007) as a colored area that is projected onto an averaged magnetic resonance image (MRI) of the brain. Gradations in this coloration indicate the probable current density.

### EEG Waveform Analysis and Correlation With Behavioral Speech Perception in Noise

Following initial EEG processing, electrodes were divided into several regions of interest (ROI). ROIs were chosen based on previous reports of optimal recording locations of CSEPs (Hämäläinen et al., 1990) and active cortical areas (i.e., temporal and parietal cortices which generally correspond to auditory and somatosensory cortices, respectively) that were identified during CDR. Analysis was performed on the following ROIs: (1) Left Temporal (LTemp) ROI (electrodes: TP7, T9, P9, TP9, T5-P7); (2) Left Parietal (LPar) ROI (electrodes: P3, P5, CP1, P1, PO7, PO3); (3) Right Parietal (RPar) ROI (electrodes: P4, P6, CP2, P2, PO8, PO4); (4) Right Temporal (RTemp) ROI (electrodes: TP8, T10, P10, TP10, T6-P8). Given that the EGI EEG recording system employs a geodesic electrode organization pattern, the above listed electrodes represent approximate 10-20 system electrode locations, as reported in Luu and Ferree (2000).

Grand average CSEPs were calculated for each ROI by first averaging ROI electrodes’ waveforms for each subject, from which latencies and amplitudes were computed. Each participant’s ROI waveform were averaged to create a grand average. Then, each participant’s ROI waveforms were plotted and latency, absolute and peak-to-peak amplitude were extracted and noted. Latency and amplitude values were then used in between-subjects statistical comparisons in order to assess the differences between groups from each ROI (using SPSS Statistical Software, version 24; IBM Corp. Released, 2015, Armonk, NY, USA). Given that EEG data were not normally distributed, non-parametric Mann-Whitney U Tests were used to compare latency and amplitude values between groups. CSEP latencies and amplitudes that were found to be significantly different between groups were correlated (using Spearman’s Rho) with participant’s speech perception scores to assess possible links between neural activity and behavioral speech perception in noise. Multiple comparisons in both between group comparisons and correlations were corrected using the False Discovery Rate correction method introduced by Benjamini and Hochberg (1995). A false discovery rate of 0.1 was determined prior to correction and then used in these calculations.

## Results

### Current Density Reconstruction (CDR) Results

**Figure 2:**
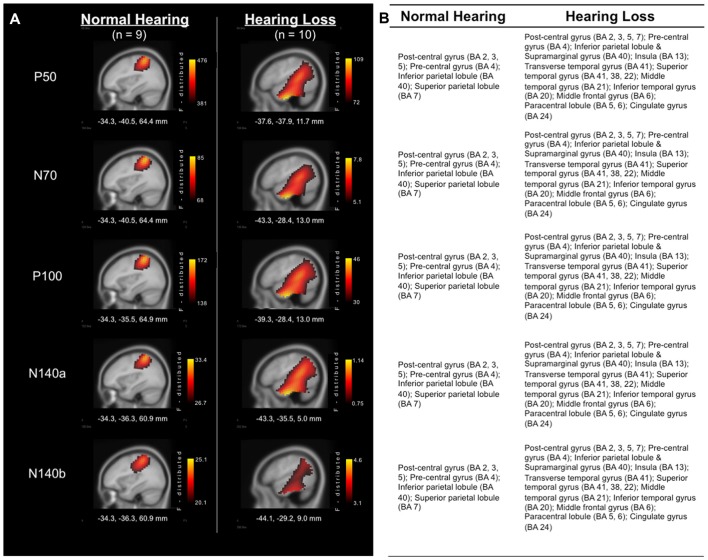
**(A)** Current density reconstructions (CDRs) of cortical activity in response to vibrotactile stimuli at the time of each of the major cortical somatosensory evoked potentials (CSEP) peaks (P50, N70, P100, N140a, N140b). Adults with NH are represented in the left column, while results from those with HL are found in the right column. Current density (i.e., Standardized Low Resolution Brain Electromagnetic Tomography (sLORETA) F-distribution) is coded by a color gradient, such that yellow represents strongest activity, with increasingly darker colors standing for weaker activity. **(B)** List of activated areas at each of the CSEP peak time points for both the NH (left) and HL (right) groups. Activations are shown for the left hemisphere.

Analysis of the sources of cortical activity, via sLORETA, yielded evidence of differences between the group of adults with NH and those with HL (see Figure 2). EEG signals were recorded from all scalp electrodes in both hemispheres and sLORETA analysis (shown in Figure 2) revealed the expected primarily contralateral source activation (i.e., left hemisphere). Of this activity, the primary activation was in post-central gyrus (i.e., primary and secondary somatosensory cortices), in Brodmann Areas (BA) 2, 3, and 5, for both groups. In addition, both groups exhibited activity in the pre-central gyrus, and inferior and superior parietal lobules (BA 4, 40, and 7, respectively). However, the HL group presented with additional activations that were not observed in the NH group in many regions that are activated during auditory processing (Figure 2B). These cortical regions included: Transverse temporal gyrus (BA 41); Superior temporal gyrus (BA 41, 22); Temporal pole (BA38); Middle temporal gyrus (BA 21); Inferior temporal gyrus (BA 20); Supramarginal gyrus (BA 40); Insula (BA 13); Middle frontal gyrus (BA 6); Paracentral lobule (BA 5, 6); and Cingulate gyrus (BA 24). These findings point to cross-modal reorganization of the auditory cortices by the somatosensory system in participants with HL only.

### CSEP Waveform Analysis

**Figure 3:**
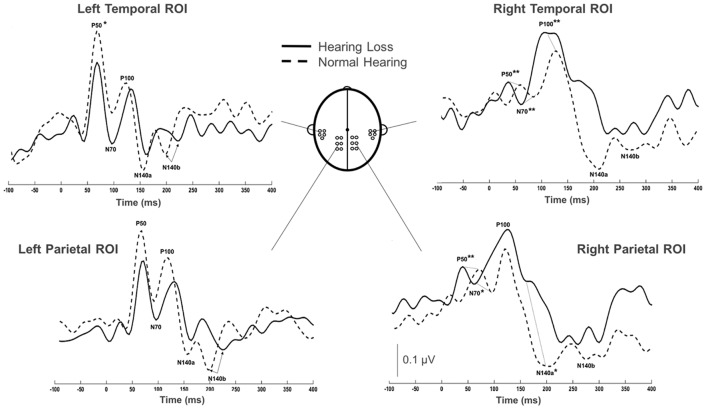
Comparison of grand averaged CSEP waveforms of the HL (solid) and NH (dashed) groups from the Left Temporal (LTemp), Left Parietal (LPar), Right Parietal (RPar) and Right Temporal (RTemp) ROIs. Significantly different CSEP peak latencies are denoted by asterisks—single asterisks indicate significance at the *p* = 0.05 level, while double asterisks highlight differences at the *p* ≤ 0.001 level. Y-axis scale (μV; bottom middle) corresponds to all waveforms.

Group grand average CSEP waveforms for the Left Temporal (LTemp), Right Parietal (RPar), and Right Temporal (RTemp) ROIs can be seen in Figure 3. Statistical comparison of CSEP peak latencies revealed significant differences in three of four regions of interest (ROI; see Table 1). In the LTemp ROI, the P50 CSEP waveform component latency was significantly earlier in the HL group (*p* = 0.01; *U* = 75.5). From the RPar ROI, the HL group’s P50 (*p* = 0.001; *U* = 82.0), N70 (*p* = 0.004; *U* = 78.5), and N140a (*p* = 0.008; *U* = 76.5) CSEP peak latencies were significantly earlier compared to those of the NH group. CSEP latencies from the RTemp ROI were also earlier in the HL group for the P50 (*p* = 0.00; *U* = 86.0), N70 (*p* = 0.00; *U* = 88.0) and P100 (*p* = 0.00; *U* = 89.0) waveform components, relative to the NH group. No difference in mean CSEP absolute or peak-to-peak amplitude was observed in any ROIs. In general, shorter peak latencies have been taken to suggest more efficient stimulus processing (and shorter latencies in the HL group are considered markers of cross-modal plasticity Buckley and Tobey, 2011; Sandmann et al., 2012; Campbell and Sharma, 2014).

**Table 1:** Mean cortical somatosensory evoked potential (CSEP) peak latencies and standard deviations for CSEP waveform components that were found to be statistically different between the hearing loss (HL) and normal hearing (NH) groups.

ROI and waveform component	Group	Mean latency (ms)	Std. deviation (ms)	95% confidence interval (upper—lower bound)	Statistic
LTemp P50	HL	57.2	18.4	43.9–70.4	0.01; 75.5
	NH	72	4.9	54.6–79.1	
RPar P50	HL	43.2	8.3	37.2–49.2	0.001; 82.0
	NH	64.4	10.8	57.1–71.7	
RPar N70	HL	66.4	10.1	59.1–73.7	0.004; 78.5
	NH	89.7	17.9	77.5–101.7	
RPar N140a	HL	139.6	26.9	120.3–158.9	0.01; 76.5
	NH	181.3	27.5	162.6–199.8	
RTemp P50	HL	45.2	8.2	39.3–51.1	0.00; 86.0
	NH	68	11.3	59.9–75.3	
RTemp N70	HL	60	10.5	52.5–67.5	0.00; 88.0
	NH	89.7	11.6	82.4–98.4	
RTemp P100	HL	96.8	11.7	88.4–105.2	0.00; 89.0
	NH	129.3	13.8	188.2–137.8	

*Latencies and standard deviations reported in milliseconds (ms)*.

### Relationship of Cross-Modal Reorganization and Functional Outcome

**Figure 4:**
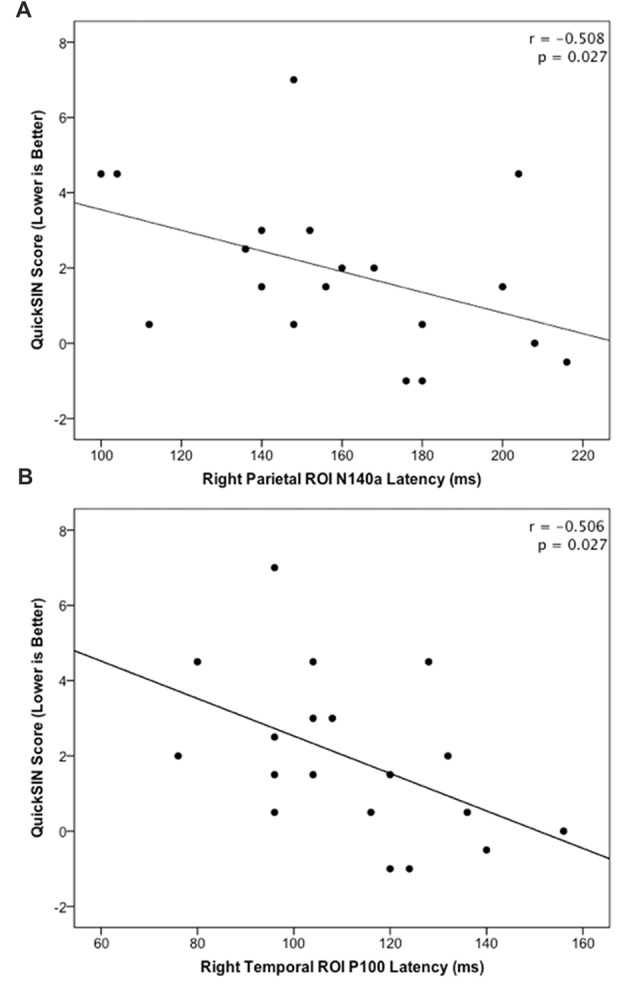
**(A)** Significant negative correlation between QuickSIN score and the N140 CSEP peak latency from the Right Parietal (RPar) ROI (*r* = −0.508; *p* = 0.027). **(B)** Significant negative correlation between QuickSIN score and the P100 CSEP peak latency from the Right Temporal (RTemp) ROI (*r* = −0.506; *p* = 0.027).

The HL and NH groups did not differ significantly in their mean QuickSIN scores (*p* = 0.079; *U* = 23.0). However, a trend was observed in these scores between groups, such that the NH group tended to score lower suggestive of better speech perception in noise (mean = 1.0; S.D. = 1.9), while the HL group tended to present with higher (worse) scores (mean = 2.75; S.D. = 2.06) consistent with previous studies (Gifford et al., 2011). In order to examine possible relationships between neural activity and behavioral performance, CSEP waveform peak latencies from the LTemp, RPar and RTemp ROIs that were significantly different between groups were correlated against QuickSIN scores. These analyses revealed two significant findings following correction for multiple comparisons (Benjamini and Hochberg, 1995). First, the peak latency of the N140a CSEP waveform component from the RPar ROI was significantly negatively correlated with QuickSIN scores (*r* = −0.508; *p* = 0.027; see Figure 4A) such that adults who showed greater difficulty perceiving speech in noise also showed earlier latencies (indicative of cross-modal reorganization). Similarly, earlier RTemp P100 CSEP peak latencies were correlated with worse speech perception in noise scores on the QuickSIN test (*r* = −0.506; *p* = 0.027; see Figure 4B). These results are consistent with previous studies of cross-modal reorganization by vision in deaf adults where earlier cortical response latencies have been associated with worse speech perception (Buckley and Tobey, 2011; Sandmann et al., 2012; Campbell and Sharma, 2014).

## Discussion

We investigated whether adults with ARHL showed evidence of somatosensory cross-modal reorganization and if this reorganization was related to behavioral speech perception in noise. CDRs showed patterns of activation in cortical regions typically associated with somatosensory processing in both HL and NH adults, but only HL adults showed additional activation of auditory processing areas, such as the STG, and association and multisensory areas (Figure 2). Additionally, CSEP waveform analysis revealed earlier peak latencies for the P50 CSEP waveform component in the LTemp ROI, the P50, N70 and N140a components in the RPar ROI, and the P50, N70 and P100 CSEP components in the RTemp ROI for the HL group, compared with the NH group (Figure 3). Finally, the latencies of the RPar, N140a and the RTemp P100 CSEP components were negatively correlated with functional performance on a clinical test of speech perception in noise (i.e., QuickSIN), suggesting that those listeners who exhibited more difficulty in speech perception showed more evidence of cross-modal somatosensory plasticity (Figure 4). Overall, our results suggest that adults with age-related, mild-moderate HL exhibit cross-modal cortical reorganization from the somatosensory modality and that such reorganization is associated with decreased speech perception in noise performance.

### Somatosensory Cross-Modal Reorganization in Mild-Moderate ARHL

Given that a basic tenet of neuroplasticity is that the brain will reorganize following sensory deprivation (Merabet and Pascual-Leone, 2010), it is not surprising that a long period of profound sensory deprivation in deafness results in somatosensory cross-modal plasticity. Indeed, several studies have shown evidence of somatosensory cross-modal reorganization in both deaf animals and humans (Sadato et al., 1996; Levänen et al., 1998; Baldwin, 2002; Auer et al., 2007; Sharma et al., 2007; Allman et al., 2009; Meredith and Lomber, 2011; Karns et al., 2012; Wong et al., 2015). However, the current study demonstrates for the first time that cross-modal reorganization of the auditory cortex by the somatosensory system occurs in humans when sensory deprivation is relatively modest, that is, in age-related mild-moderate HL.

### Possible Mechanisms of Somatosensory Cross-Modal Plasticity in Hearing Loss

The majority of studies on cross-modal reorganization in HL have been investigated in the visual modality (for a review, see Glick and Sharma, 2017). This relationship may seem more intuitive than that of the auditory and somatosensory systems with respect to HL, because as hearing ability decreases, individuals often rely heavily on visual input to enhance functions such as speech understanding. This enhanced dependence on vision has been localized to areas of the auditory cortex in animal and human studies (Lomber et al., 2010; Stropahl et al., 2015; Stropahl and Debener, 2017). Similarly, it is possible that deficiencies in auditory input could lead to increased reliance on somatosensory information to improve behavioral performance (e.g., Shore et al., 2016). The close proximity and anatomical and physiological connections between the auditory and somatosensory systems also support the possibility of their cross-modal interaction. For instance, anatomical proximity and convergence of auditory and somatosensory neuronal pathways occurring at subcortical, thalamo-cortical and cortico-cortical levels, and somatosensory processing occurring in primary and higher-order auditory cortices, has been demonstrated in animal studies (Lindsley et al., 1999; Schroeder and Foxe, 2002; Fu et al., 2003; Brosch et al., 2005; Lakatos et al., 2005, 2007; Hackett et al., 2007; Zeng et al., 2012; Kok and Lomber, 2017) and human brain imaging investigations (Foxe et al., 2000, 2002; Gobbelé et al., 2003; Caetano and Jousmäki, 2006; Lütkenhöner and Klein, 2007). In addition, behavioral studies in humans have revealed interactions between the auditory and somatosensory systems (Jousmäki and Hari, 1998; Spence et al., 1998; Merat et al., 1999; Schürmann et al., 2004). Since these connections are already in place, weakening of the function of the auditory system, due to diminished or degraded input, could lead to unmasking of latent multisensory connections (Soto-Faraco and Deco, 2009). Such a process may underlie appropriation of cortical regions typically dominated by auditory processing by other sensory modalities, such as somatosensation.

In addition to anatomical features that set up the possibility of somatosensory to auditory interactions, sound and vibration fundamentally consist of the same physical process (i.e., oscillation), although they are typically propagated through different media—sound through air and vibration through solids (von Bèkèsy, 1959; Levänen et al., 1998; Soto-Faraco and Deco, 2009). Unlike the visual and auditory systems, somatosensory-auditory interaction may be driven by the physical similarity of the stimuli processed by these sensory systems and because neurons in these systems respond to inputs with an overlapping range of frequencies (i.e., ~5–300 Hz; Levänen et al., 1998; Harrington and Hunter Downs, 2001). Given that the temporal patterns and neural response frequencies of the auditory and somatosensory systems show significant overlap, it is likely that the auditory subcortical and cortical neurons could accurately encode somatosensory input (Levänen et al., 1998). Thus, given a reduction of the incoming auditory signal, it may be possible for the somatosensory system to utilize auditory cortical space for vibrotactile, and other, somatosensory processing. In fact, it has been shown that a reduction in auditory activity leads to increases in somatosensory activity in subcortical structures (Dehmel et al., 2008; Shore et al., 2008; Shore, 2011; Zeng et al., 2011), which could possibly continue into cortical regions. In all, because of the high degree of interconnectivity between the various cortical and subcortical levels of these two sensory systems, and because of the similarity of auditory and vibrotactile stimuli, decreased activity in either modality might lead to increased activity in the other (Soto-Faraco and Deco, 2009; Zeng et al., 2012). Additionally, this type of process could lead to more efficient processing of somatosensory input resulting in the decreased latencies described in the current study and consistent with previous studies which describe a greater ease of processing of somatosensory information in auditory cortex (Levänen et al., 1998; Foxe et al., 2000; Levänen and Hamdorf, 2001; Schroeder et al., 2001).

### Functional Implications of Cross-Modal Plasticity in Adults With Hearing Loss

Cross-modal plastic cortical changes may underlie changes in functional behavior in adults who develop HL. Previous studies in deaf adults and children fitted with cochlear implants have shown that visual cross-modal plasticity is negatively correlated with speech perception (Doucet et al., 2006; Sandmann et al., 2012; Campbell and Sharma, 2016). In the present study, CSEP latencies were also significantly negatively correlated with speech perception in noise ability in the RTemp and RPar ROIs (Figure 4). That is, adults who had more difficulty with behavioral speech perception showed greater cross-modal reorganization by the somatosensory modality. While the participants in the current study exhibited the early-stages of ARHL according to its clinical definition (i.e., hearing thresholds ≥25 dBHL), in actuality HL is a continuous variable. As such, a mild case of HL may not lead to behavioral scores that were statistically different from the norm, as exhibited by the lack of difference in scores between the NH and HL groups. However, the correlational analysis presented in the current results suggest that greater degrees of cross-modal reorganization are associated with poorer speech perception in noise scores. Interestingly, consistent with the present study, there is some evidence to support the notion that the right temporal cortices are more susceptible to functional reorganization, although the reason for this effect remains to be determined (Finney et al., 2001; Sandmann et al., 2012; Campbell and Sharma, 2013, 2014, 2016; Cardin et al., 2013; Lin et al., 2014; Kim et al., 2016; Peelle and Wingfield, 2016; Sharma et al., 2016; Shiell et al., 2016).

Speech perception is a multisensory process, requiring input from audition, vision and somatosensation (e.g., Bernstein and Benoît, 1996). Therefore, as suggested by the current results, deficiencies in one sensory modality may lead to increased reliance on other modalities’ contributions to speech perception. For instance, several studies have linked aspects of speech perception to somatosensory processing. In one study, Huang et al. (2017) recently showed that the speech perception in noise abilities of cochlear implant users were enhanced by presenting low frequency vibrotactile stimuli to participants’ fingers simultaneously with auditory stimuli, vs. auditory stimulation alone. Gick and Derrick (2009) showed that untrained participants with NH integrated tactile information into their perception of auditory speech. In that study, participants were more likely to perceive a phoneme as aspirated (e.g., “p” vs. “b”) when an inaudible air puff was presented to their skin as they listened to these phonemes. This propensity was taken as evidence of speech-related auditory-tactile integration. Recent investigations have indicated that deaf individuals can use vibrotactile information to differentiate same-sex talkers based on the frequency content of their distinct voices, as well as various musical instruments due to their unique timbre (Russo et al., 2012; Ammirante et al., 2013). Thus, it appears that the somatosensory system is able to utilize vibrotactile information alone to accurately decode complex speech and speech-like information. Furthermore, Ito et al. (2009) demonstrated that systematically deforming the facial skin in a speech-like manner altered the perception of simultaneously presented auditory phonemes. Also, studies performed in deaf cats indicate that vocalizations may be influenced by somatosensory input—i.e., an auditory feedback loop that is primed by somatosensory perception (Hubka et al., 2015). Finally, studies have also shown evidence of activation of motor cortices associated with speech production during speech listening (Fadiga et al., 2002; Watkins et al., 2003; Meister et al., 2007) supporting the view that one’s own speech *production*, a process heavily mediated by the somatosensory system, can inform speech *perception* (Liberman et al., 1967; Liberman and Mattingly, 1985; Callan et al., 2010). Taken together with the present results, the above studies suggest that the somatosensory system plays an important role in speech perception. It follows, then, that a diminished or degraded auditory signal, as in HL, could lead to increased dependence on somatosensory input during difficult listening situations. This reliance could, in turn, be a driving factor in somatosensory cross-modal reorganization of the auditory cortex in individuals with HL, even in its earliest stages.

In response to findings similar to those reported above, a recent study from our laboratory suggests that intervention may have the potential to reverse functional consequences of cortical plastic changes. Sharma et al. (2016) documented visual and somatosensory cross-modal reorganization in a patient with single-sided deafness. Following cochlear implantation of the deaf ear, this patient showed a complete reversal of somatosensory cross-modal plasticity and significantly improved auditory processing (as evidenced by localization and better speech perception). Thus, it appears that appropriate treatment of auditory deficiency may lead to reversal of cortical reorganization. This notion is in line with recent evidence in deaf cats that suggests that auditory cortices are not completely recruited by other sensory modalities and that re-introduction of auditory input stimulates the preserved auditory neural function (Land et al., 2016). Future studies should address whether cross-modal reorganization of the auditory cortices like those shown in the current study can be reversed following intervention.

In addition to declines in performance on behavioral tasks, such as speech perception in noise, recent studies have linked ARHL to cognitive decline including all cause dementia and Alzheimer’s disease (Lin et al., 2011a,b, 2013; Lin, 2011, 2012). It has been speculated that the increased cognitive load resulting from the recruitment of additional neural networks to supplement listening in adults with ARHL results in effortful listening and may accelerate cognitive decline (Cardin, 2016; Peelle and Wingfield, 2016). Given that cross-modal recruitment reflects a fundamental change in cortical resource allocation (Campbell and Sharma, 2013), future investigations should also endeavor to determine the relationship between the altered neural circuitry underlying cross-modal plastic changes and cognitive load.

In conclusion, adults with early-stage mild-moderate ARHL showed evidence of cross-modal plasticity between the somatosensory and auditory systems. Furthermore, adults with ARHL who showed greater difficulty processing speech in difficult listening situations (i.e., noise) showed greater evidence of somatosensory crossmodal reorganization, suggesting that functional dependence on intact modalities may serve as an aid to communication in real-world situations. Thus, even mild sensory deficit has fundamental impacts on the organization and functioning of the auditory cortex. Our findings may have implications for understanding neuroplastic changes in ARHL and its future treatment.

## Ethics Statement

This study was carried out in accordance with the recommendations of the Belmont Report as reviewed by the Institutional Review Board of the University of Colorado Boulder with written informed consent from all subjects or their guardians. Additionally, all children ages seven and above provided written assent prior to participating in the study. All subjects gave written informed consent/assent in accordance with the Declaration of Helsinki. The protocol was approved by the Institutional Review Board of the University of Colorado Boulder.

## Author Contributions

GC and AS contributed equally to the conception and design of the work, data analysis and interpretation, and critical revision and final approval of the article. GC collected all data and drafted the article initially.

## Conflict of Interest Statement

The authors declare that the research was conducted in the absence of any commercial or financial relationships that could be construed as a potential conflict of interest.
